# Supplementary material for: Mucosal IgA to pre-fusion F protein predicts protection from RSV infection in a high burden setting
Source: Nat Commun. 2026 Aug 26;17:9056. doi: 10.1038/s41467-026-77315-4 (PMC13518992; doi:10.1038/s41467-026-77315-4)
Supplement: Supplementary file 2 [file 41467_2026_77315_MOESM2_ESM.pdf]

nature portfolio

Corresponding author(s): David Hodgson

Last updated by author(s): Jun 29, 2026

Reporting Summary

Nature Portfolio wishes to improve the reproducibility of the work that we publish. This form provides structure for consistency and transparency in reporting. For further information on Nature Portfolio policies, see our Editorial Policies and the Editorial Policy Checklist.

Statistics

For all statistical analyses, confirm that the following items are present in the figure legend, table legend, main text, or Methods section.

n/a	Confirmed	
☐	☒	The exact sample size (*n*) for each experimental group/condition, given as a discrete number and unit of measurement
☐	☒	A statement on whether measurements were taken from distinct samples or whether the same sample was measured repeatedly
☐	☒	The statistical test(s) used AND whether they are one- or two-sided<br>*Only common tests should be described solely by name; describe more complex techniques in the Methods section.*
☐	☒	A description of all covariates tested
☐	☒	A description of any assumptions or corrections, such as tests of normality and adjustment for multiple comparisons
☐	☒	A full description of the statistical parameters including central tendency (e.g. means) or other basic estimates (e.g. regression coefficient) AND variation (e.g. standard deviation) or associated estimates of uncertainty (e.g. confidence intervals)
☒	☐	For null hypothesis testing, the test statistic (e.g. *F*, *t*, *r*) with confidence intervals, effect sizes, degrees of freedom and *P* value noted<br>*Give P values as exact values whenever suitable.*
☐	☒	For Bayesian analysis, information on the choice of priors and Markov chain Monte Carlo settings
☐	☒	For hierarchical and complex designs, identification of the appropriate level for tests and full reporting of outcomes
☐	☒	Estimates of effect sizes (e.g. Cohen's *d*, Pearson's *r*), indicating how they were calculated

Our web collection on statistics for biologists contains articles on many of the points above.

Software and code

Policy information about availability of computer code

Data collection	Data collection did not involve custom software. RSV PCR testing and antibody assays were performed using standard laboratory equipment and protocols as described in the Methods.
Data analysis	All statistical analyses and Bayesian hierarchical modelling were performed in Stan (version 0.5.3) using Hamiltonian Monte Carlo sampling via cmdstanr in R version 4.2.3. Custom model code is publicly available at https://github.com/ccgh-idd/cop-transvir-rsv.

For manuscripts utilizing custom algorithms or software that are central to the research but not yet described in published literature, software must be made available to editors and reviewers. We strongly encourage code deposition in a community repository (e.g. GitHub). See the Nature Portfolio guidelines for submitting code & software for further information.

Data

Policy information about availability of data

All manuscripts must include a data availability statement. This statement should provide the following information, where applicable:

- Accession codes, unique identifiers, or web links for publicly available datasets
- A description of any restrictions on data availability
- For clinical datasets or third party data, please ensure that the statement adheres to our policy

The anonymised individual-level data required to reproduce the analyses and fit the models are publicly available at https://github.com/ccgh-idd/cop-transvir-rsv. Raw data from the TRANSVIR study contain potentially patient-identifiable information and are therefore not publicly available, in line with the terms of ethical

approval (LSHTM ethics ref: LEO project ID 22556; MRCG Ethics Committee) and participant consent. Access to these data may be requested by contacting the corresponding author.

Research involving human participants, their data, or biological material

Policy information about studies with human participants or human data. See also policy information about sex, gender (identity/presentation), and sexual orientation and race, ethnicity and racism.

Reporting on sex and gender	Sex was recorded at enrolment as a binary biological variable (male/female) based on self-report. Sex-disaggregated data are reported in the cohort characteristics. Gender identity was not collected as a separate variable, as this was not relevant to the primary research questions of this immunological study.
Reporting on race, ethnicity, or other socially relevant groupings	The study was conducted in a single community in The Gambia. Ethnicity was not collected as a study variable as it was not relevant to the research questions, and the cohort does not have sufficient ethnic diversity to support ethnicity-stratified analyses.
Population characteristics	Participants were members of households recruited from a peri-urban community in The Gambia. The cohort included individuals across the full age spectrum from infants to older adults. Key demographic characteristics including age, sex, and household size are reported in a previous publication, Jarju, S., Wenlock, R.D., Danso, M. et al. High SARS-CoV-2 incidence and asymptomatic fraction during Delta and Omicron BA.1 waves in The Gambia. Nat Commun 15, 3814 (2024). https://doi.org/10.1038/s41467-024-48098-3
Recruitment	Households were recruited through community engagement and door-to-door approach at the study site. Participation was voluntary and written informed consent was obtained from all adult participants and from parents/guardians of children. Assent was additionally obtained from older children where appropriate.
Ethics oversight	The study received ethical approval from the Gambia Government/MRC Joint Ethics Committee and the London School of Hygiene & Tropical Medicine Ethics Committee. The study was conducted in accordance with the Declaration of Helsinki.

Note that full information on the approval of the study protocol must also be provided in the manuscript.

Field-specific reporting

Please select the one below that is the best fit for your research. If you are not sure, read the appropriate sections before making your selection.

☒ Life sciences ☐ Behavioural & social sciences ☐ Ecological, evolutionary & environmental sciences

For a reference copy of the document with all sections, see nature.com/documents/nr-reporting-summary-flat.pdf

Life sciences study design

All studies must disclose on these points even when the disclosure is negative.

Sample size	No formal sample size calculation was performed for the correlates of protection analysis, which was secondary to the primary epidemiological study. The sample size was determined by the parent household cohort study (Jarju, Wenlock et al.), which was powered to detect RSV incidence. All individuals with available paired serological samples and confirmed RSV infection or confirmed non-infection status were included in the correlate-of-protection analysis (n=342).
Data exclusions	Participants were excluded from the correlate-of-protection analysis if they had insufficient paired serological samples or missing covariate data. Individuals with subsequent reinfection episodes (n=4) were excluded from the primary model due to the added complexity of characterising re-exposure risk and post-infection immune modification; these are described separately in Supplementary Methods. No other data exclusions were made.
Replication	This is an observational cohort study; experimental replication in the traditional sense was not applicable. The robustness of findings was assessed through multiple pre-specified sensitivity analyses, including alternative exposure models and alternative biomarker definitions, all of which returned consistent conclusions regarding the relative performance of candidate correlates.
Randomization	Randomization was not applicable. This was an observational household cohort study; participants were not allocated to experimental groups.
Blinding	Laboratory staff performing antibody assays were blinded to participants' infection status. The statistical modelling and correlate-of-protection analyses were performed after infection classification was finalised; no blinding of analysts to outcomes was applied at the modelling stage, as the Bayesian hierarchical framework jointly estimates infection status and antibody kinetics simultaneously.

Reporting for specific materials, systems and methods

We require information from authors about some types of materials, experimental systems and methods used in many studies. Here, indicate whether each material, system or method listed is relevant to your study. If you are not sure if a list item applies to your research, read the appropriate section before selecting a response.

Materials & experimental systems

n/a	Involved in the study
☐	☒ Antibodies
☒	☐ Eukaryotic cell lines
☒	☐ Palaeontology and archaeology
☒	☐ Animals and other organisms
☐	☒ Clinical data
☒	☐ Dual use research of concern
☒	☐ Plants

Methods

n/a	Involved in the study
☒	☐ ChIP-seq
☒	☐ Flow cytometry
☒	☐ MRI-based neuroimaging

Antibodies

Antibodies used

PE-conjugated mouse anti-human IgG secondary antibody (Abcam, ab99761) and PE-conjugated goat anti-human IgA secondary antibody (Abcam, ab99909), used for detection in the Luminex multiplex immunoassay. For the live virus neutralisation assay, Alexa488-conjugated anti-RSV antibody MAB88262 (1:3000) was used for immunostaining, with DAPI for nuclear counterstaining. Nirsevimab was used as a positive control in neutralisation assays.

Validation

Secondary antibodies were commercial validated reagents used according to manufacturer's instructions (Abcam). Luminex assay performance was validated through use of a standard curve to convert MFI values to arbitrary units, enabling interpolation across plates and runs. The relevance of the Luminex biomarkers as antigenic targets was independently validated against live virus neutralisation, with 97 serum and 97 mucosal samples spanning the full antibody titre distribution tested in the microneutralisation assay. RSV BT2a viral stocks were titrated by immunostaining plaque assay prior to use.

Clinical data

Policy information about clinical studies

All manuscripts should comply with the ICMJE guidelines for publication of clinical research and a completed CONSORT checklist must be included with all submissions.

Clinical trial registration

The study protocol was registered at ClinicalTrials.gov (NCT05952336).

Study protocol

The TRANSVIR household cohort study design has been previously described in detail (ref 56). Ethics approval was granted by the joint Gambia Government and Medical Research Council Unit The Gambia (MRCG) Ethics Committee, and the London School of Hygiene & Tropical Medicine Ethics Committee (LEO project ID 22556).

Data collection

Participants were followed for 12 months with weekly combined throat and nose flocked swabs collected into RNAprotect cell reagent. Symptom data were collected at each weekly sampling point covering the preceding 7 days. Venous blood for serum separation and nasal lining fluid via synthetic absorptive matrix (SAM) strip were collected at enrolment and 6-monthly thereafter (three timepoints total). Households with symptomatic participants were additionally swabbed at unscheduled visits with contemporaneous RT-PCR testing. Households ranged from 6 to 10 members, including at least one adult and one child. Written informed consent was obtained from all participants aged over 18, with thumbprint consent where required; assent was obtained from those aged 12–18; and parental or guardian consent for those under 12.

Outcomes

The primary outcome for the correlates-of-protection analysis was PCR-confirmed or serologically-inferred RSV infection during the 12-month follow-up period. Separate infection episodes were defined as a new PCR-positive result with no prior positive for the same virus in the preceding 28 days. Symptomatic versus asymptomatic status was a secondary outcome, defined by self-reported symptoms in the 7 days preceding each swab. No severe or hospitalised RSV cases occurred during the study period.

Plants

Seed stocks

N/A

Novel plant genotypes

N/A

Authentication

N/A
