# Supplementary material for: Mucosal IgA to pre-fusion F protein predicts protection from RSV infection in a high burden setting
Source: Nat Commun. 2026 Aug 26;17:9056. doi: 10.1038/s41467-026-77315-4 (PMC13518992; doi:10.1038/s41467-026-77315-4)
Supplement: Supplementary file 3 [file 41467_2026_77315_MOESM3_ESM.pdf]

nature portfolio

Peer Review File

Mucosal IgA to pre-fusion F protein predicts protection from RSV infection in a high burden setting

Corresponding Author: Dr David Hodgson

This file contains all reviewer reports in order by version, followed by all author rebuttals in order by version.

Version 0:

Reviewer comments:

Reviewer #1

(Remarks to the Author)
With interest I read the manuscript named "Mucosal IgA to pre-fusion F protein predicts protection from RSV infection in a high burden setting" by Hodgson and colleagues in which they describe the results of a household study in the Gambia in which extensive sampling for RSV and associated antibodies was performed to provide more insight in correlates of protection against RSV disease. Their work is crucial in understanding RSV disease and aid development of preventive strategies. The study is well conducted and clearly described in great detail. I do have some follow-up questions/suggestions.

Major
1. The finding that just 17.3% of PCR positive infections were symptomatic is intriguing and against the current believe that most RSV infections present symptomatically. Can the authors provide more detail about these asymptomatic versus symptomatic cases? Think of:
o Are Ct values different (high Ct in asymptomatic group? Potentially false positives at the limit of detection? Disease that has already resolved?)
o Age group differences?
o Differences in perception of symptoms? Country/continent related differences in how symptoms are perceived since the discussion mentioned that studies in other African countries also found a large proportion of asymptomatic patients contradicting high-income country literature?
2. Lines 101-102: How were separate episodes defined? These four patients with multiple episodes are of special interest since their immunity was either not complete or not long lasting? How long were the episodes spaced apart in time and is there any detail about their antibody dynamics as compared to 1) other RSV patients with just one episode 2) the rest of the cohort.
3. The failed Narsyn trial (RCT with daily intranasal droplets of palivizumab; PMID: 38192587) did not observe a protective effect of the intervention by administration of antibodies on the mucosa. How does this relate to the results of your study? Palivizumab is an IgG (which should not be on the mucosa, and is more potent to postF than against Pre-F which might explain the failed protective effect? Might a monocloncal pre-F IgA (or IgG like nirsevimab) administered nasally be a viable strategy? What path for next steps do the authors foresee
4. The findings based on the neutralization assay that young infants require much higher mucosal IgA titers as compared to adults to achieve 50% inhibition is interesting and is likely the result of (a lack of) maturation as the authors mentioned. Have the authors considered avidity testing to quantify this mechanism (I am not sure whether this is even possible or validated for RSV IgA/IgG..)

Minor
5. Lines 46-48: Abrysvo is also registered for adults and MRESVIA (the RSV vaccine of Moderna) has also been approved for adults.
6. Line 206: remove the dot after 80%
7. Line 210: "high" is not the right attribute for an AUC of 0.65-0.72. "Stable"?

(Remarks on code availability)

Reviewer #2

(Remarks to the Author)
General comments:
Thank you for the opportunity to review this interesting manuscript, which compares mucosal and serum antibody titres against major RSV proteins in their ability to predict RSV infection in a household cohort in The Gambia. The paper makes use of a rich dataset of serological and clinical data collected longitudinally from a well-defined population. The manuscript is informative, well-written and provides useful insights relevant to understanding RSV immunological responses and developing preventive strategies. I cannot comment on the details of serological assays, but from an epidemiological perspective the analysis appears to be well done. I have a few minor comments for consideration below.

Minor comments:
1. Intro, L60-67: it would be helpful to clarify what is meant by “high/low transmission”, “high/low burden”, “high/low resource” settings. I understand the intent in this paragraph is to highlight the possibility that RSV exposure throughout life could influence immunity, and that differences in transmission patterns could lead to different patterns of immunity in different settings. But these terms seem to be used interchangeably here to contrast a setting like The Gambia with a place like, say, the UK. Transmission levels in the UK are likely lower than in The Gambia, particularly in younger age groups, but I wouldn’t describe RSV transmission in high-income countries as “low”. Burden is also lower (but again, I wouldn’t characterize it as “low”), but is a function of many factors, including transmission levels, demographics, risk factor prevalence, access to care etc.

2. In the results, I suggest adding some clearer information regarding the age distribution of participants. I infer from some of the age groupings presented in the results and supplementary information that the number of participants in key age groups currently targeted by immunization (particularly infants and the elderly) was small. I would think it’s worth mentioning this as a potential limitation in the discussion, since the results presented may not fully reflect immunological responses in those key age groups.

(Remarks on code availability)
I have looked at the code made available through a github repository. I have not attempted to run the code, but from the repository, it appears to be well documented and includes the code to generate the data in the paper as well as output files of figures and tables.

Reviewer #3

(Remarks to the Author)

Thank you for the opportunity to review this manuscript. In the study, the authors collected immunological RSV data from participants in The Gambia over 12 months. Using these data, the authors constructed a Bayesian hierarchical modelling framework to jointly estimate RSV-specific antibody kinetics following infection and to quantify an age-specific correlate of protection against RSV infection, for different biomarkers.

This is a nicely written paper that makes an important contribution to our understanding of correlates of protection against RSV infection, a field in which evidence is currently quite limited. I found that the supplementary methods were particularly important for understanding the hierarchical modelling methodology. I have only a few comments.

Main comments

A key part of the hierarchical model is that the number of infections in a household is used as a proxy for exposure to infection (plus some small level of external infection pressure). Because household size is an integer (would be helpful if you indicated the median & range of household sizes), does this limit the analysis because there would only be (some limited number of) categorical levels for the infection exposure? Have you tried to test whether assuming that more of the exposure pressure comes from some background level of infection changes outcomes?

Looking at Figure 1 panel C – it looks like in the groups of individuals who were infected, do the infections always occur around the Oct 2021 mark? If an infection occurred either before or after the RSV season (I’m not sure how strongly seasonal RSV dynamics are in The Gambia), does that mean the trajectory for that individual is still marked as orange?

Minor comments

1. Introduction para 1, lines 45-48. Additional products mRESVIA and clesrovimab now also approved.

2. Line 301-304, you mention “Vaccines already licensed for use in younger infants may not provide the same protection if used on other age groups (e.g. school-age children) when relying solely on infant-derived protection correlations; age-specific correlates should be established rather than extrapolated” – we don’t currently have RSV vaccines for use in younger infant populations, so I wasn’t sure what you were referring to here?

3. Supplement line 177 – assume you mean log-linear waning?

4. Some minor typos in the supplementary methods – please check carefully. E.g. line 187 “log10 scale”, “given”. Also lines 246, 248.

(Remarks on code availability)

Reviewer #4

(Remarks to the Author)
This manuscript by Hodgson et al describes a household cohort study in The Gambia, following a large group of people during 12 months. This is an extensive and thoroughly performed study with interesting outcomes, eg emphasizing the importance of mucosal immunity and showing the high number of asymptomatic infections in this population. I believe this work is original and of high quality. These results are very relevant for further clinical studies of RSV vaccines or antivirals. The manuscript is well organized and written, and I have no major concerns with the contents.

I do have some minor comments and thoughts.

• Repeatedly some figures are not correctly referenced (eg Supp. Fig. 6,7 and 8 and Fig 4/5). Please check all figure references. The order of the supp. Figs should be adapted accordingly.
• Next to IgA, serum IgG could be present in the nasal lining fluid, especially during infections. Were the nasal samples checked for this?
• Fig 1B is confusing, so the Y-axis is participant ID ordered by time of infection?
• Supplementary fig1. Why is there a difference in width of bars? Panels are labeled c and d, this labeling is not reflected in the caption.
• Line 118: do you mean both RSV A and B?
• Supplementary fig 4: no background histograms (orange or gray) can be seen. Line 795, solid pink or green line. Figure is labeled with 'A', this letter can be omitted.
• Supp fig 5. Figure is labeled with 'A', this letter can be omitted. This figure should not be referenced in line 159, it does not fit there. It is referenced correctly in the next paragraph.
• Line 165: 'evaluate' instead of 'evaluating'
• Fig. 3, some fonts are too small (eg the 'infected, protected, NA' in 3d, or the age in Fig4).
• Supp. Fig 8: legend is not correct (blue line in the dots).
• Line 272: discrepancy doesn’t seem too illogical, as mucosal IgA is abundant at site of infection compared to serum IgG. Serum IgG is probably a better correlate for protection against symptomatic or severe infection compared to mucosal IgA, could this be detected in this study? Do mucosal IgA levels correlate with protection against severe (or at least symptomatic) disease?
• Line 282: is it really needed to define protection against infection as most infections are asymptomatic?
• Line 417: typo

(Remarks on code availability)

Version 1:

Reviewer comments:

Reviewer #1

(Remarks to the Author)
I thank the authors for their extensive work to answer the comments raised by the reviewers. I am happy with their explanation and the changes made to the manuscript. I do not have any follow-up questions or concerns.

(Remarks on code availability)

Reviewer #3

(Remarks to the Author)
Thank you for providing such detailed and thoughtful responses to my comments (and those of the other reviewers). I am satisfied that my concerns have been addressed in the revised version of the manuscript.

(Remarks on code availability)

Open Access This Peer Review File is licensed under a Creative Commons Attribution 4.0 International License, which permits use, sharing, adaptation, distribution and reproduction in any medium or format, as long as you give appropriate credit to the original author(s) and the source, provide a link to the Creative Commons license, and indicate if changes were made.
In cases where reviewers are anonymous, credit should be given to 'Anonymous Referee' and the source.
The images or other third party material in this Peer Review File are included in the article's Creative Commons license, unless indicated otherwise in a credit line to the material. If material is not included in the article's Creative Commons license and your intended use is not permitted by statutory regulation or exceeds the permitted use, you will need to obtain permission directly from the copyright holder.
To view a copy of this license, visit https://creativecommons.org/licenses/by/4.0/

REVIEWER COMMENTS

Reviewer #1 (Remarks to the Author):

With interest I read the manuscript named "Mucosal IgA to pre-fusion F protein predicts protection from RSV infection in a high burden setting" by Hodgson and colleagues in which they describe the results of a household study in the Gambia in which extensive sampling for RSV and associated antibodies was performed to provide more insight in correlates of protection against RSV disease. Their work is crucial in understanding RSV disease and aid development of preventive strategies. The study is well conducted and clearly described in great detail. I do have some follow-up questions/suggestions.

Major

1. The finding that just 17.3% of PCR positive infections were symptomatic is intriguing and against the current believe that most RSV infections present symptomatically. Can the authors provide more detail about these asymptomatic versus symptomatic cases?

Think of:

o Are Ct values different (high Ct in asymptomatic group? Potentially false positives at the limit of detection? Disease that has already resolved?)
o Age group differences?

We thank the reviewer for raising this important point. To directly address the concern about whether asymptomatic detections may represent false positives near the limit of detection, or infections that have already resolved, we have generated a new supplementary figure (Supplementary Methods Figure 2) showing Ct values stratified by age and symptom status across all PCR-confirmed RSV infections. Symptomatic and asymptomatic infections show broadly overlapping Ct value distributions across all age groups, with no systematic elevation of Ct values in the asymptomatic group that would suggest spurious low-level positives near the assay threshold (Ct ≤37). Both groups show a modest increase in Ct with age, consistent with higher pre-existing immunity in older individuals attenuating peak viral replication, rather than asymptomatic infections being detected at the limit of detection. Supplementary Methods Figure 2 therefore provides reassurance that the high asymptomatic fraction reflects genuine productive infections across the age spectrum rather than a detection artefact.

o Differences in perception of symptoms? Country/continent related differences in how symptoms are perceived since the discussion mentioned that studies in other African countries also found a large proportion of asymptomatic patients contradicting high-income country literature?

We thank the reviewer for this important point. We agree this warrants explanation. The high asymptomatic fraction is consistent with findings from independent African community-based cohorts; e.g. the PHIRST study in South Africa (67% asymptomatic RSV) and a coastal Kenya household study, suggesting this reflects a genuine epidemiological signal in African community settings rather than a reporting or ascertainment artefact. This likely reflects two complementary factors: first, community-based symptom-agnostic surveillance designs capture the full infection spectrum unlike some hospital-based retrospective studies which inherently select for

symptomatic cases; second, the high background burden of respiratory symptoms in peri-urban African settings may reduce participant reporting of mild RSV-associated symptoms such as cough and nasal congestion. The detailed analysis of symptomatic fraction by age, Ct value, and setting is reported in depth in the companion epidemiological paper (Jarju, Wenlock et al., ref 19), to which we direct readers for full discussion. We have added the following sentence to the Discussion on lines 272–276:

"This substantial undetected transmission suggests RSV circulates far more widely than symptomatic surveillance indicates, likely reflecting both the full-spectrum design of community-based surveillance and a high background respiratory symptom burden in these settings rather than differences. A full epidemiological analysis is reported in Jarju et al"

2. Lines 101-102: How were separate episodes defined? These four patients with multiple episodes are of special interest since their immunity was either not complete or not long lasting? How long were the episodes spaced apart in time and is there any detail about their antibody dynamics as compared to 1) other RSV patients with just one episode 2) the rest of the cohort.

We thank the reviewer for this question. Separate infection episodes were defined as a new PCR-positive result for the same virus with no prior positive in the preceding 28 days, following the definition established in the companion epidemiological paper (Jarju, Wenlock et al., ref 19). We have added the following sentence to the Methods section (after the paragraph describing PCR confirmation, before the serology methods on line 447–44)):

"Separate RSV infection episodes were defined as a new PCR-positive result with no prior positive for the same virus in the preceding 28."

Regarding the four individuals with multiple PCR-confirmed RSV episodes, we have added a brief descriptive summary figure in the Supplementary Methods. We note that given only four reinfected individuals and only three PCR-confirmed RSV-B infections across the entire cohort, it is not statistically meaningful to draw inferences about differences in antibody kinetics between reinfected and single-episode individuals, or between RSV-A and RSV-B infected individuals. We have update the manuscript to says on lines 569–573:

"Correlate of protection models were fitted using each individual's first (primary) RSV infection only; the small number of individuals with subsequent infections (n=4) were excluded from this analysis due to the added model complexity required to characterise re-exposure risk and post-infection immune modification. These subsequent infections are described in Supplementary Methods."

And have included a Figure showing the characteristic of subsequent infections in the Supplementary Methods Figure 3.

3. The failed Narsyn trial (RCT with daily intranasal droplets of palivizumab; PMID: 38192587) did not observe a protective effect of the intervention by administration of antibodies on the mucosa. How does this relate to the results of your study? Palivizumab is an IgG (which should not be on the mucosa, and is more potent to postF than against Pre-F which might explain the failed protective effect? Might a monocloncal

pre-F IgA (or IgG like nirsevimab) administered nasally be a viable strategy? What path for next steps do the authors foresee

We thank the reviewer for raising the Narsyn trial, which is relevant to our findings but not previously addressed.

The Narsyn trial tested daily intranasal palivizumab as passive mucosal prophylaxis in late preterm infants and was found to be ineffective at preventing RSV. We offer three mechanistic perspectives on this failure. First, our findings identify mucosal IgA (not IgG) to PreF as the strongest correlate of protection (AUC 0.72 vs 0.65), and secretory IgA has structural properties that exogenously administered IgG cannot replicate at mucosal surfaces. Second, palivizumab preferentially targets the post-fusion conformation, whereas PreF was the superior antigenic target in our data. Third, and importantly, the Narsyn authors themselves identify short mucosal IgG half-life due to mucociliary clearance as the most plausible explanation for failure, and notably showed that re-engineering palivizumab as secretory IgA paradoxically reduced murine efficacy; cautioning against the straightforward conclusion that isotype switching alone would be sufficient. We agree that a nasally delivered anti-PreF strategy with addressed mucosal pharmacokinetics remains a rational next step, and whether the potency and extended half-life of nirsevimab could overcome mucosal clearance limitations by intranasal delivery is an open question warranting investigation. We propose the following addition to the Discussion on lines 388–397 :

"Beyond active vaccination, our findings are also relevant to passive mucosal prophylaxis strategies using monoclonal antibodies. The failure of the Narsyn trial of intranasal palivizumab[52] is instructive alongside our findings. Palivizumab is an IgG preferentially targeting the post-fusion F conformation, whereas we identify mucosal IgA to PreF as the strongest correlate of protection. Secretory IgA has structural properties suited to the mucosal environment that exogenous IgG cannot replicate, and the Narsyn authors identify short mucosal IgG half-life via mucociliary clearance as the most plausible explanation for failure.[52] Future mucosal passive prophylaxis strategies should address both antigenic target (PreF) and mucosal pharmacokinetics; whether the potency and extended half-life of nirsevimab could overcome mucosal clearance limitations via intranasal delivery warrants future investigation."

4. The findings based on the neutralization assay that young infants require much higher mucosal IgA titers as compared to adults to achieve 50% inhibition is interesting and is likely the result of (a lack of) maturation as the authors mentioned. Have the authors considered avidity testing to quantify this mechanism (I am not sure whether this is even possible or validated for RSV IgA/IgG..)

We thank the reviewer for this suggestion. We agree that avidity maturation is a highly plausible mechanism underlying the age-dependent titre-protection relationship we observe, and that avidity testing (e.g. by chaotropic ELISA) is technically feasible for RSV-specific IgA and IgG, though to our knowledge not yet well validated specifically for mucosal IgA responses to RSV antigens.

We would expect that immunological maturation of the antibody response with age would indeed be accompanied by increased avidity, consistent with the general principle of affinity maturation through repeated antigen exposure and germinal centre cycling. However, we would also highlight that avidity is unlikely to be the sole explanatory mechanism: differences in epitope targeting and the breadth of the antibody repertoire directed against distinct antigenic sites on the F protein may be equally important, as has been described for other respiratory viruses such as influenza. Disentangling the relative contributions of avidity, epitope specificity, and breadth would require a dedicated mechanistic investigation using structural and functional assays beyond the binding antibody measurements employed here.

We consider this an important and worthwhile direction for follow-up mechanistic work, but believe it falls outside the scope of the current study, which is fundamentally an immuno-epidemiological modelling analysis designed to identify population-level correlates of protection rather than to characterise the underlying mechanisms of antibody function.

Minor

5. Lines 46-48: Abrysvo is also registered for adults and MRESVIA (the RSV vaccine of Moderna) has also been approved for adults.

Thank you for this correction. We have updated the text to reflect that Abrysvo is approved for both adults and older adults, and have added mRESVIA (Moderna) as an additionally approved RSV vaccine for adults and provided reference to the seminal papers.

6. Line 206: remove the dot after 80%

Thank you for catching this. The dot after 80% has been removed.

7. Line 210: “high” is not the right attribute for an AUC of 0.65-0.72. “Stable”?

Thank you for the suggestion. We agree that "high" was not an appropriate descriptor for an AUC in this range. We have replaced it with "moderate".

Reviewer #2 (Remarks to the Author):

General comments:
Thank you for the opportunity to review this interesting manuscript, which compares mucosal and serum antibody titres against major RSV proteins in their ability to predict RSV infection in a household cohort in The Gambia. The paper makes use of a rich dataset of serological and clinical data collected longitudinally from a well-defined population. The manuscript is informative, well-written and provides useful insights relevant to understanding RSV immunological responses and developing preventive strategies. I cannot comment on the details of serological assays, but from an epidemiological perspective the analysis appears to be well done. I have a few minor comments for consideration below.

Minor comments:
1. Intro, L60-67: it would be helpful to clarify what is meant by “high/low transmission”, “high/low burden”, “high/low resource” settings. I understand the intent in this paragraph is to highlight the possibility that RSV exposure throughout life could influence immunity, and that differences in transmission patterns could lead to different patterns of immunity in different settings. But these terms seem to be used interchangeably here to contrast a setting like The Gambia with a place like, say, the UK. Transmission levels in the UK are likely lower than in The Gambia, particularly in younger age groups, but I wouldn’t describe RSV transmission in high-income countries as “low”. Burden is also lower (but again, I wouldn’t characterize it as “low”), but is a function of many factors, including transmission levels, demographics, risk factor prevalence, access to care etc.

We thank the reviewer for this helpful clarification. We agree that "high/low transmission", "high/low burden" and "high/low resource" were used imprecisely and potentially interchangeably in this paragraph, and that none of these terms accurately characterises RSV transmission in high-income settings as simply "low". We have revised the relevant sentences to more precisely reflect what we mean: that cumulative lifetime RSV exposure is likely greater in settings like The Gambia due to higher transmission intensity, particularly in early childhood, and that this, rather than burden or resource level per se, is what may drive distinct patterns of immunity, on lines 66–76:

"Moreover, the majority of existing immunological data derive from high-income settings, despite LMICs bearing over 95% of RSV-attributable mortality.14 This geographical imbalance is important because cumulative RSV exposure across the life course likely differs substantially between settings: in high-transmission peri-urban and rural communities in LMICs, repeated childhood exposure from an early age may lead to distinct patterns of immune boosting, antibody persistence and protective immunity compared to settings where transmission intensity is comparatively lower.13–15 Understanding immune dynamics in high-transmission, high-mortality settings is therefore important both for characterising how natural exposure shapes population immunity and for designing vaccination strategies for the populations at greatest risk."

2. In the results, I suggest adding some clearer information regarding the age distribution of participants. I infer from some of the age groupings presented in the results and supplementary information that the number of participants in key age groups

currently targeted by immunization (particularly infants and the elderly) was small. I would think it’s worth mentioning this as a potential limitation in the discussion, since the results presented may not fully reflect immunological responses in those key age groups.

We thank the reviewer for this observation. The reviewer is correct that the cohort was not designed to oversample the age groups currently targeted by licensed RSV immunisation programmes; infants under 5 years (n=41, 11.7%) and adults ≥50 years (n=25, 7.2%) — and that the correlate-of-protection estimates for these groups therefore carry greater uncertainty than for school-age children and working-age adults. The age distribution of PCR-confirmed and serologically-defined infections across these groups is already shown in Supplementary Figure S1, which clearly illustrates the relatively small numbers of infected individuals in the <5 and ≥50 year age groups that underpin the age-stratified analyses. We have added the following sentence to the Limitations paragraph to flag this explicitly on lines 403–407:

"The cohort was not designed to oversample age groups currently targeted by licensed RSV immunisation programmes: infants under 5 years (n=41) and adults aged ≥50 years (n=25) were underrepresented relative to the broader population, as illustrated in Supplementary Figure S1, and the age-stratified correlate-of-protection estimates for these groups should therefore be interpreted with caution.”

Reviewer #2 (Remarks on code availability):

I have looked at the code made available through a github repository. I have not attempted to run the code, but from the repository, it appears to be well documented and includes the code to generate the data in the paper as well as output files of figures and tables.

Reviewer #3 (Remarks to the Author):

Thank you for the opportunity to review this manuscript. In the study, the authors collected immunological RSV data from participants in The Gambia over 12 months. Using these data, the authors constructed a Bayesian hierarchical modelling framework to jointly estimate RSV-specific antibody kinetics following infection and to quantify an age-specific correlate of protection against RSV infection, for different biomarkers.

This is a nicely written paper that makes an important contribution to our understanding of correlates of protection against RSV infection, a field in which evidence is currently quite limited. I found that the supplementary methods were particularly important for understanding the hierarchical modelling methodology. I have only a few comments.

Main comments

A key part of the hierarchical model is that the number of infections in a household is used as a proxy for exposure to infection (plus some small level of external infection pressure). Because household size is an integer (would be helpful if you indicated the median & range of household sizes), does this limit the analysis because there would only be (some limited number of) categorical levels for the infection exposure? Have you tried to test whether assuming that more of the exposure pressure comes from some background level of infection changes outcomes?

We thank the reviewer for this insightful question. We have added the median (IQR) and range of household size to the Supplementary Methods in Figure 4. The reviewer is correct that the number of infected household members, H_i, is a small non-negative integer bounded by household size, which limits the granularity of this exposure intensity variable; however, the logistic exposure model still outputs a continuous exposure probability $\pi_i$even though its input is essentially discrete.

Following the reviewer's suggestion to test whether assuming more exposure pressure comes from background/external infection changes our findings, we fitted an alternative version of the exposure model in which individual exposure probability was fixed at a single constant value, estimated across the whole cohort, with no dependence on the number of infected household members. This effectively removes household infection burden as a driver of exposure risk and instead attributes all exposure to a uniform background level of infection pressure, providing a direct test of the household-size-dependent exposure assumption in our base-case model. We have added the following sentence to the Methods describing the exposure model on lins 588–591:

“As a sensitivity analysis, we additionally fitted the model with exposure probability fixed at a constant value, independent of the number of infected household members, to assess the robustness of our findings to this assumption.”

We have also added the corresponding results to the Results on lines 238–245:

"We additionally tested whether our assumption that exposure probability scales with the number of infected household members affected our findings, by fitting a sensitivity analysis with exposure probability fixed at a constant value independent of household infection burden. While absolute AUC values shifted under this alternative exposure assumption, the relative ranking of biomarkers and the identification of PreF mucosal IgA as the strongest correlate of protection remained consistent. Therefore, despite substantial differences in infection classification and exposure assumptions, our key findings regarding the relative performance of candidate correlates remained consistent across all scenarios tested."

This sensitivity analysis is illustrated in a new Supplementary Results Figure 5.1, comparing biomarker performance between the household-size-dependent exposure model and the fixed-exposure-probability model. We thank the reviewer again for raising this point, which we believe has strengthened confidence in the robustness of our correlate-of-protection findings.

Looking at Figure 1 panel C – it looks like in the groups of individuals who were infected, do the infections always occur around the Oct 2021 mark? If an infection occurred either before or after the RSV season (I'm not sure how strongly seasonal RSV dynamics are in The Gambia), does that mean the trajectory for that individual is still marked as orange?

We thank the reviewer for this careful observation. To clarify: almost all first RSV infections in this cohort, both PCR-confirmed and serologically-inferred, occurred within the period between the enrollment and 6-month bleeds (stated in the Results), which corresponds to the October 2022–March/April 2023 window shown in Figure 1A. Few infections were detected in the second 6-month period of follow-up (6–12 months), consistent with a single, relatively well-defined RSV epidemic wave during the study period, in keeping with previously reported RSV seasonality in The Gambia.

Minor comments

1. Introduction para 1, lines 45-48. Additional products mRESVIA and clesrovimab now also approved.

Thank you for this correction. We have updated the text to reflect that mRESVIA (Moderna) as an additionally approved RSV vaccine for adults and clesrovimab for infants along with the references in the revised manuscript.

2. Line 301-304, you mention "Vaccines already licensed for use in younger infants may not provide the same protection if used on other age groups (e.g. school-age children) when relying solely on infant-derived protection correlations; age-specific correlates should be established rather than extrapolated" – we don't currently have RSV vaccines for use in younger infant populations, so I wasn't sure what you were referring to here?

We thank the reviewer for catching this imprecision. The intended point was a hypothetical caution rather than a statement about current licensing, since, as the reviewer notes, no RSV

vaccine is currently licensed for direct active immunisation of young infants. We have revised the sentence to make this hypothetical framing explicit on lines 343–348 of the revised manuscript.

"This raises an important consideration for future paediatric RSV vaccine development: should an active vaccine for infants be developed and its correlate of protection established using infant-derived data, our findings suggest this correlate may not generalise to other age groups, such as school-age children, due to age-dependent differences in the titre-protection relationship. Age-specific correlates should therefore be established directly within each target population rather than extrapolated from one age group to another."

3. Supplement line 177 – assume you mean log-linear waning?

We thank the reviewer for catching this. We confirm the waning process is indeed log-linear (linear decay of the log10 titre value over time), and have corrected the text at this line to state "log-linear waning".

4. Some minor typos in the supplementary methods – please check carefully. E.g. line 187 “log10 scale”, “given”. Also lines 246, 248.

We thank the reviewer for their careful reading and apologise for these typographical errors. We have corrected "log10 scale" and "given" at line 187, as well as the errors at lines 246 and 248.

Reviewer #4 (Remarks to the Author):

This manuscript by Hodgson et al describes a household cohort study in The Gambia, following a large group of people during 12 months. This is an extensive and thoroughly performed study with interesting outcomes, eg emphasizing the importance of mucosal immunity and showing the high number of asymptomatic infections in this population. I believe this work is original and of high quality. These results are very relevant for further clinical studies of RSV vaccines or antivirals. The manuscript is well organized and written, and I have no major concerns with the contents.

We thank the reviewer for their assessment of our manuscript and for recognising the relevance of these findings for future RSV vaccine and antiviral development.

I do have some minor comments and thoughts.

• Repeatedly some figures are not correctly referenced (eg Supp. Fig. 6,7 and 8 and Fig 4/5). Please check all figure references. The order of the supp. Figs should be adapted accordingly.

We apologise for these inconsistencies and thank the reviewer for flagging them. We have carefully checked all in-text figure and supplementary figure references throughout the manuscript, corrected the misreferences identified (including Supplementary Figures 6, 7 and 8, and Figures 4 and 5), and reordered the supplementary figures so that their numbering follows the sequence in which they are first cited in the text.

• Next to IgA, serum IgG could be present in the nasal lining fluid, especially during infections. Were the nasal samples checked for this?

We thank the reviewer for this point. Due to resource constraints, we tested for the predominant immunoglobulin subclass in each compartment, serum IgG and mucosal IgA, rather than measuring all isotypes across both compartments. We agree that some serum IgG is likely present in nasal lining fluid, reflecting passive transudation from the circulation. This phenomenon has been well described for SARS-CoV-2; mucosal immunity has shown strong correlation between serum and nasal IgG (consistent with transudation), in contrast to weak correlation between serum and nasal IgA, indicating that mucosal IgA responses are predominantly locally generated rather than transudated.[1] We would therefore expect the live virus neutralisation activity measured in our nasal lining fluid samples to reflect a combination of both locally-produced IgA and transudated serum IgG, which may partly explain the comparatively lower correlation we observed between neutralisation and mucosal IgA alone.

[1] Bladh O, Marking U, Havervall S et al. ***Mucosal immune responses following a fourth SARS-CoV-2 vaccine dose.*** *The Lancet Microbe, 2023; 4e488*

• Fig 1B is confusing, so the Y-axis is participant ID ordered by time of infection?

Yes, this interpretation is correct. As this was unclear, we have revised the Figure 1B to include a more descriptive axis.

• Supplementary fig1. Why is there a difference in width of bars? Panels are labeled c and d, this labeling is not reflected in the caption.

We thank the reviewer for catching this. The difference in bar width was an artefact of automatic formatting in ggplot rather than a feature of the data; we have corrected this so that bar width is now consistent throughout each group. We have also updated the figure caption to correctly reflect the panel labelling (C and D).

• Line 118: do you mean both RSV A and B?

We thank the reviewer for this question, and apologise for the lack of clarity. We confirm that the kinetics analysis at line 118 includes both RSV-A and RSV-B infections, pooled together, rather than RSV-A alone. We have corrected the text at line 118 to state this explicitly. We have also added the following sentence to the Limitations paragraph of the Discussion on line 412–417, acknowledging that pooling serotypes is a simplification with potential consequences for interpretation, given the very small number of RSV-B infections (n=3) identified in this cohort:

"We pooled RSV-A and RSV-B infections when modelling antibody kinetics due to the small number of PCR-confirmed RSV-B infections (n=3) in this cohort, which precluded serotype-specific kinetic modelling. This pooling assumes similar antibody kinetics across serotypes and may conflate subtype-specific dynamics; larger studies with greater RSV-B representation are needed to determine whether kinetics differ meaningfully by serotype."

• Supplementary fig 4: no background histograms (orange or gray) can be seen. Line 795, solid pink or green line. Figure is labeled with 'A', this letter can be omitted.

We apologize for this oversight. The caption was incorrect due to rearrangement of figures, and the legend in the text (referring to background histograms in orange/gray, and the line color) had not been updated accordingly. The "A)" label was a legacy artifact from an earlier version of the figure. This has now been corrected: the caption and in-text description now accurately reflect the current figure, and the redundant "A)" label has been removed.

• Supp fig 5. Figure is labelled with 'A'; this letter can be omitted. This figure should not be referenced in line 159, it does not fit there. It is referenced correctly in the next paragraph.

We apologise for this oversight. We have removed the "A" label from Supplementary Figure 5, and have corrected the erroneous reference at line 159, removing it from that location; the figure is correctly referenced in the following paragraph as the reviewer notes.

• Line 165: 'evaluate' instead of 'evaluating'

We have now corrected this typo.

• Fig. 3, some fonts are too small (eg the 'infected, protected, NA' in 3d, or the age in Fig4).

We thank the reviewer for flagging this. We have increased the font size of the labels in Figure 3 and the age axis labels in Figure 4 to improve legibility.

• Supp. Fig 8: legend is not correct (blue line in the dots).

We thank the reviewer for catching this. We have corrected the legend in Supplementary Figure 8 to accurately reflect the point styling used in the figure.

• Line 272: discrepancy doesn't seem too illogical, as mucosal IgA is abundant at site of infection compared to serum IgG. Serum IgG is probably a better correlate for protection against symptomatic or severe infection compared to mucosal IgA, could this be detected in this study? Do mucosal IgA levels correlate with protection against severe (or at least symptomatic) disease?

We thank the reviewer for this point. The reviewer's suggested explanation aligns with clinical trial data for licensed RSV interventions (nirsevimab and maternal vaccination), which act via serum IgG and are highly effective against hospitalisation and severe disease.

However, our study lacks the power to test this hypothesis. Our correlate-of-protection model defines the outcome as any RSV infection (PCR-confirmed or serologically-inferred, symptomatic or asymptomatic combined), rather than symptomatic or severe disease specifically. This was a necessary design choice given that only 14 of 81 PCR-confirmed infections (17.3%) were symptomatic, and no severe or hospitalised cases occurred in this community-based cohort, numbers too small to support a separate correlate-of-protection analysis restricted to symptomatic or severe outcomes. We therefore interpret our finding as indicating that mucosal PreF IgA is the strongest correlate of protection against any *RSV infection, rather than specifically against symptomatic or severe disease. We would also note, however, that preventing infection necessarily prevents any downstream symptomatic or severe disease that infection might otherwise cause; an intervention capable of blocking infection at the mucosal entry point therefore has clear relevance to severe disease prevention, even if our study cannot directly quantify a correlate of that specific endpoint.*

• Line 282: is it really needed to define protection against infection as most infections are asymptomatic?

We thank the reviewer for this point. We agree it's worth justifying. Although most infections were asymptomatic, defining protection against infection itself still matters: asymptomatic individuals continue to shed virus and transmit to vulnerable contacts, so interventions that block infection altogether offer transmission-reducing benefits that symptomatic or hospitalisation endpoints alone would miss. We have added the following sentence after line 282:

"Although most RSV infections in our cohort were asymptomatic, defining correlates of protection against infection itself remains relevant given that asymptomatic infections still enable onward transmission to vulnerable contacts."

• Line 417: typo

We have now fixed this typo in the revised manuscript.
